# MicroRNA Profiles in Normotensive and Hypertensive South African Individuals

**DOI:** 10.3389/fcvm.2021.645541

**Published:** 2021-04-16

**Authors:** Don M. Matshazi, Cecil J. Weale, Rajiv T. Erasmus, Andre P. Kengne, Saarah F. G. Davids, Shanel Raghubeer, Stanton Hector, Glenda M. Davison, Tandi E. Matsha

**Affiliations:** ^1^South African Medical Research Council/Cape Peninsula University of Technology Cardiometabolic Health Research Unit, Department of Biomedical Sciences, Faculty of Health and Wellness Sciences, Cape Peninsula University of Technology, Cape Town, South Africa; ^2^Division of Chemical Pathology, Faculty of Health Sciences, National Health Laboratory Service and Stellenbosch University, Cape Town, South Africa; ^3^Non-communicable Diseases Research Unit, South African Medical Research Council, Cape Town, South Africa; ^4^Department of Medicine, University of Cape Town, Cape Town, South Africa

**Keywords:** hypertension, microRNA, blood pressure, cardiovascular, non-coding, sub-Saharan Africa

## Abstract

Hypertension has a complex pathogenesis and symptoms appear in advanced disease. Dysregulation of gene expression regulatory factors like microRNAs has been reported in disease development. Identifying biomarkers which could help understand the pathogenesis and prognosis of hypertension is essential. The study's objective was to investigate microRNA expression profiles according to participant blood pressure status. Next generation sequencing was used to identify microRNAs in the whole blood of 48 body mass index-, smoking- and age-matched normotensive (*n* = 12), screen-detected hypertensive (*n* = 16) and known hypertensive (*n* = 20) female participants. Quantitative reverse transcription polymerase chain reaction was used to validate the next generation sequencing findings in a larger, independent sample of 84 men and 179 women. Using next generation sequencing, 30 dysregulated microRNAs were identified and miR-1299 and miR-30a-5p were the most significantly differentially expressed. Both microRNAs were upregulated in known hypertensives or screen-detected hypertensives compared to the normotensives. Kyoto Encyclopedia of Genes and Genomes pathway enrichment analysis indicated possible involvement of platelet activation, calcium signaling and aldosterone synthesis pathways. Further validation of miR-1299 and miR-30a-5p using quantitative reverse transcription polymerase chain reaction confirmed sequencing results while yielding new findings. These findings demonstrate microRNA dysregulation in hypertension and their expression may be related to genes and biological pathways essential for blood pressure homeostasis.

## Introduction

The 8th report released by the Joint National Committee on Prevention, Detection and Evaluation of High Blood Pressure describes hypertension (HPT) as the persistent elevation of blood pressure (BP) above the 140/90 mmHg threshold (1, 2). Despite efforts to understand the pathogenesis of the condition, HPT remains a leading public health concern affecting both developed and developing countries (3, 4). It has been identified as one of the most important modifiable risk factors for cardiovascular disease, renal disease, and stroke, and accounts for over 10 million deaths throughout the world annually (5–7). In 90–95% of HPT patients, the cause is unknown, and is thus termed primary or essential HPT (8, 9). However, research has demonstrated the involvement of genetic and environmental factors in the development of HPT. The influence of epigenetic factors such as deoxyribonucleic (DNA) methylation and histone modification on the pathogenesis of HPT has been a subject of intense research, with several important conclusions being made along the way (10). However, there is a paucity of research regarding microRNAs (miRNAs) in the context of HPT.

MiRNAs are a group of small, endogenous, non-coding ribonucleic (RNA) sequences that are 17–25 base pairs long (11). These molecules are involved in gene expression regulation at the post-transcriptional level. This is achieved by binding to the 3′untranslated region of complementary messenger RNA (mRNA) molecules and inhibiting translation into protein or inducing mRNA degradation (12). MiRNAs are present in almost every cell and disturbances in their regulation are usually associated with disease processes, including HPT (13, 14). Herein, we investigated the miRNA profiles in South African individuals with normal BP, as well as those presenting with known or screen-detected HPT.

## Materials and Methods

### Ethics Statement

This investigation was based on the Cape Town Vascular and Metabolic Health (VMH) study, which was approved by the Research Ethics Committees of the Cape Peninsula University of Technology (CPUT) and Stellenbosch University (respectively, NHREC: REC−230 40−014 and N14/01/003). Ethical approval was also obtained for this cross-sectional sub-study from the CPUT Health and Wellness Sciences Research Ethics Committee (CPUT/HW-REC 2019/H7). The study was conducted as per the provisions of the Declaration of Helsinki. All procedures were explained to the participants in their language of choice. Once the participants fully understood their participation, they signed informed consent forms to allow the collection of blood and anthropometric data.

### Study Design and Procedures

Data collection and procedures have been described previously (15). Briefly, participants underwent anthropometric and BP measurements, as well as oral glucose tolerance tests (OGTT). Anthropometric measurements for each participant were taken three times and the average reported. BP was measured according to the World Health Organization (WHO) guidelines (16), using a semi-automatic digital BP monitor (Omron M6 comfort-preformed cuff BP Monitor, China) on the right arm in a sitting position and at rest for at least 10 min. Three BP readings were taken at 3-min intervals and the lowest systolic BP and corresponding diastolic BP-values were used. Participants were grouped into three categories based on; the use of anti-hypertensive medication as known HPT, BP measurement of 140/90 mm Hg or greater as screen-detected HPT and normal BP measurement (<140/90 mm Hg) as normotensive. Body Mass Index (BMI) was calculated as weight per square meter (kg/m^2^), where kg was the participant's weight in kilograms and m^2^, the square of their height.

The following biochemical parameters were analyzed at an ISO 15189 accredited Pathology practice (PathCare Reference Laboratory, Cape Town, South Africa): glycated hemoglobin (HbA1c) by High Performance Liquid Chromatography (BioRad Variant Turbo, BioRad, Hercules, CA, USA); serum insulin by a paramagnetic particle chemiluminescence assay (Beckman DXI, Beckman Coulter, South Africa); serum cotinine by Competitive Chemiluminescent (Immulite 2000, Siemens, Munich, Germany); plasma glucose by enzymatic hexokinase method (Beckman AU, Beckman Coulter, Brea, CA, USA); total cholesterol (TC); high density lipoprotein cholesterol (HDL-c) by enzymatic immunoinhibition—end point (Beckman AU, Beckman Coulter, Brea, CA, USA); triglycerides (TG) by glycerol phosphate oxidase-peroxidase, end point (Beckman AU, Beckman Coulter, Brea, CA, USA); low density lipoprotein cholesterol (LDL) by enzymatic selective protection—end point (Beckman AU, Beckman Coulter, Brea, CA, USA); and ultrasensitive C-reactive protein (CRP) by Latex Particle Immunoturbidimetry (Beckman AU, Beckman Coulter, Brea, CA, USA). In addition, blood samples were collected in a Tempus RNA tube (ThermoFisher Scientific, Waltham, MA, USA) and stored at −80°C for total RNA extraction and analysis.

### RNA Isolation

Total RNA, including miRNA, was isolated from whole blood using the MagMax for Stabilized Blood RNA isolation kit (ThermoFisher Scientific) according to manufacturer's instructions. The concentration and purity of each RNA extract was determined using a NanoDrop One spectrophotometer. Total RNA extracts with 260/280 values between 1.8 and 2.0, and concentrations >20 ng/μl were used for microRNA sequencing (miRNA-seq) using next generation sequencing (NGS) and quantitative reverse transcription PCR (RT-qPCR).

### MicroRNA Sequencing

This was conducted on total RNA samples from 48 female participants representing three different HPT statuses. The inclusion of females only in this part of the study was to avoid introducing potential sources of variation due to gender effect in an already small cohort. Small RNA library construction, deep sequencing, and data processing were performed at Arraystar Inc., Rockville, USA as previously described by Matsha et al. (15). Briefly, the total RNA of each sample was used to prepare the miRNA sequencing library as follows: (1) 3′-adapter ligation with T4 RNA ligase 2 (truncated); (2) 5′-adapter ligation with T4 RNA ligase; (3) complementary DNA (cDNA) synthesis with RT primer; (4) PCR amplification; (5) extraction and purification of ~130–150 bp PCR amplified fragments (correspond to ~15–35 nt small RNAs) from the polyacrylamide gel electrophoresis gel. The Agilent 2100 Bioanalyzer was used to quantify completed libraries, thereafter DNA fragments were denatured with 0.1 M sodium hydroxide to generate single-stranded DNA molecules, then captured on Illumina flow cells, amplified *in situ*, and finally sequenced for 51 cycles on the Illumina HiSeq system according to the manufacturer's instructions. Raw sequences were generated as clean reads from the Illumina HiSeq using real-time base calling and quality filtering. The clean reads that passed the quality filter were processed to remove adaptor sequences as the trimmed reads. The trimmed reads (length ≥ 15 nt) were aligned to the human pre-miRNA in miRBase 21, using NovoAlign software. The miRNA expression levels were measured and normalized as transcripts per million of total aligned miRNA reads.

### Gene Ontology and Functional Enrichment Analysis

The Gene Ontology (GO) analysis was performed to describe gene and gene product attributes (http://www.geneontology.org). The ontology covers three domains: Biological Process, Cellular Component and Molecular Function. Commonly predicted gene targets were subjected to functional analysis using Kyoto Encyclopedia of Genes and Genomes (KEGG). A conservative Fisher's exact-test and false discovery rate method were used to calculate the targeted pathways.

### Validation of NGS miRNA Expression Results

To confirm the expression of miRNAs, the validation of NGS results was performed on total RNA from an independent sample of 263 male and female participants randomly selected from an existing database and 48 females on which NGS had been conducted. MiRNAs were converted to cDNA using the TaqMan MicroRNA Reverse Transcription Kit according to the manufacturer's protocol (Life Technologies, USA). The miRNA expression levels were assessed using TaqMan miRNA Assay primers on the QuantStudio 7 Flex real-time PCR instrument (Life Technologies, USA) analyzer. In order to determine miRNA expression in each sample and between two groups, the 2^−ΔCt^ and 2^−ΔΔCt^ (17), respectively, were used and normalized using miR-16-5p as the endogenous control. The suitability of miR-16-5p as an endogenous control in RT-qPCR was assessed and confirmed, as there was minimal variation in its expression in normotensive and hypertensive participants.

### Statistical Analysis

Data were analyzed using R statistical software version 3.2.2 (The R Foundation for Statistical Computing, Vienna, Austria) and TIBCO Statistica version 13.5.0.17 (TIBCO Software Inc., California, USA). The Shapiro-Wilk W-test was employed to determine whether the data were normally distributed, based on probability thresholds of *p* > 0.1. Continuous variables were summarized as mean and standard deviation (SD) when normally distributed, while median, and 25th and 75th percentiles were used for skewed variables, whilst categorical variables were reported as counts and percentages. When comparing groups, for continuous variables, the analysis of variance (ANOVA) was used for normally distributed data; Kruskal Wallis-H test with Dunn *post-hoc*-test was used for skewed data, whilst the chi-square-test was used for categorical variables. Multivariable regression analysis was conducted to investigate the possible effects of these differences in baseline characteristics on the expression of miRNAs in screen-detected and known HPT. Various models were used, with variations to the crude model being used to analyse the effect or relationship of a baseline characteristic with miRNA expression in HPT. All comparisons were made with the normotensive group as the reference. A *p*-value < 0.05 was used to characterize statistically significant results. MicroRNAs with fold changes ≥1.3, and *p*-values ≤ 0.1 were selected as the differentially expressed miRNAs. Novel miRNAs were predicted using miRDeep.

## Results

### General Participant Characteristics

Of the 1988 VMH survey participants, 311 (227, 73.0% female) were selected for inclusion into this sub-study. Of these, 48 (all female) took part in the NGS part of the study while an additional 263 randomly selected male and female participants were included in the RT-qPCR validation study. The distribution of the NGS and RT-qPCR participants by BP status is shown in Table 1. The 48 women in the NGS sample included 20 with known HPT, 16 with screen-detected HPT and 12 normotensives, whilst the validation sample included 106 known hypertensives, 52 screen-detected hypertensives and 105 normotensives. The expected differences by status for HPT in the cardiovascular risk profile were apparent across the two sub-samples (Table 1).

**Table 1:** Characteristics of the participants, based on hypertension status.

	**Next generation sequencing sample**				**Validation sample (RT-qPCR)**			
	**Normotensive, *n* = 12**	**Screen-detected HPT, *n* = 16**	**Known HPT, *n* = 20**	***p*-value**	**Normotensive, *n* = 105**	**Screen-detected HPT, *n* = 52**	**Known HPT, *n* = 106**	***p*-value**
Female, *n* (%)	12 (100%)	16 (100%)	20 (100%)	-	57 (54.29)	36 (69.23)	86 (81.13)	<0.001
Male, *n* (%)	-	-	-	-	48 (45.71)	16 (30.77)	20 (18.87)	
Age (years)	49.6 ± 9.3	52.8 ± 7.1	56.1 ± 7.7	0.086	40 ± 15.32	51.12 ± 13.43	61.1 ± 10.6	<0.001
Body mass index (kg/m^2^)	29.1 ± 8.1	30.6 ± 9.0	32.3 ± 6.4	0.509	25.08 ± 6.45	28.71 ± 7.96	30.85 ± 7.06	<0.001
Waist circumference (cm)	87.5 ± 16.2	92.0 ± 22.1	97.9 ± 11.4	0.226	81.99 ± 13.71	91.32 ± 16.71	95.86 ± 14.75	<0.001
Hip circumference (cm)	101.7 ± 17.7	106.1 ± 18.4	108.8 ± 14.2	0.504	97.55 ± 12.69	103.76 ± 15.3	106.05 ± 13.85	<0.001
Waist to hip Ratio	0.86 ± 0.06	0.86 ± 0.09	0.90 ± 0.06	0.139	0.84 ± 0.07	0.88 ± 0.08	0.90 ± 0.08	<0.001
Systolic blood pressure (mmHg)	113.4 ± 14.5	147.7 ± 22.2	144.7 ± 27.7	<0.001	118.77 ± 12.95	149.12 ± 19.96	148.50 ± 23.81	<0.001
Diastolic blood pressure (mmHg)	74.8 ± 11.9	90.6 ± 14.8	89.7 ± 17.7	0.018	74.95 ± 10.47	97.19 ± 12.32	89.11 ± 13.56	<0.001
Fasting blood glucose (mmol/L)	6.18+3.87	7.50+4.58	8.28+4.27	0.413	4.87 ± 1.43	5.55 ± 2.72	6.70 ± 3.49	<0.001
2-h fasting glucose	8.63 ± 4.39	9.84 ± 6.53	12.83 ± 4.54	0.172	5.69 ± 2.8	6.8 ± 4.37	7.74 ± 4.47	0.002
HbA1c (%)	6.28 ± 1.60	7.10 ± 2.82	7.77 ± 2.67	0.276	5.79 ± 1.14	6.19 ± 1.49	6.70 ± 1.74	<0.001
Fasting insulin (mIU/L)	5.88 ± 3.49	7.89 ± 3.89	15.44 ± 8.66	<0.001	6.81 ± 6.65	8.03 ± 6.24	11.14 ± 14.33	0.011
Diabetes mellitus, *n* (%)	5 (41.7)	6 (37.5)	13 (65.0)	0.093	7 (6.7)	7 (13.7)	35 (33.3)	<0.001
Triglycerides-S (mmol/L)*	1.12 (0.86–1.64)	1.26 (1.00–1.50)	1.74 (1.43–3.31)	0.008	1.05 (0.72; 1.42)	1.28 (0.9; 1.67)	1.40 (1.05; 1.83)	<0.001
Total cholesterol (mmol/L)	5.93 ± 1.14	5.66 ± 1.12	5.93 ± 1.23	0.757	4.75 ± 1.18	5.13 ± 0.97	5.42 ± 1.04	<0.001
LDL-cholesterol (mmol/L)	3.76 ± 1.09	3.48 ± 0.97	3.96 ± 1.06	0.402	2.86 ± 1	3.13 ± 0.96	3.37 ± 0.91	0.001
HDL-cholesterol (mmol/L)	1.57 ± 0.50	1.48 ± 0.59	1.17 ± 0.21	0.032	1.36 ± 0.41	1.35 ± 0.38	1.37 ± 0.34	0.984
usCRP (mg/L)	6.32 ± 8.79	9.73 ± 13.20	11.00 ± 6.89	0.44	7.32 ± 13.51	6.24 ± 7.09	7.24 ± 14.03	0.871
Serum cotinine (ng/mL)*	10.0 (10.0–22.5)	209.5 (10.0–261.0)	99.4 (10.0–195.5)	0.146	137 (10; 265.5)	10 (10; 287)	10 (10; 135.75)	0.002
JIS MetS criteria	4 (33.33)	9 (56.25)	18 (90.00)	0.014	22 (21.15)	21 (41.18)	61 (58.65)	<0.001

Values presented as mean ± SD unless marked with an asterisk*, in which case the median and (25th-75th percentiles) are reported. The Kruskal-Wallis-test and analysis of variance (ANOVA) were used to compare the median and mean baseline characteristics, respectively, across blood pressure groups. SD, standard deviation; usCRP, ultrasensitive CRP; MetS, Metabolic Syndrome.

### NGS miRNA Expression Profiling

**Figure 1:**
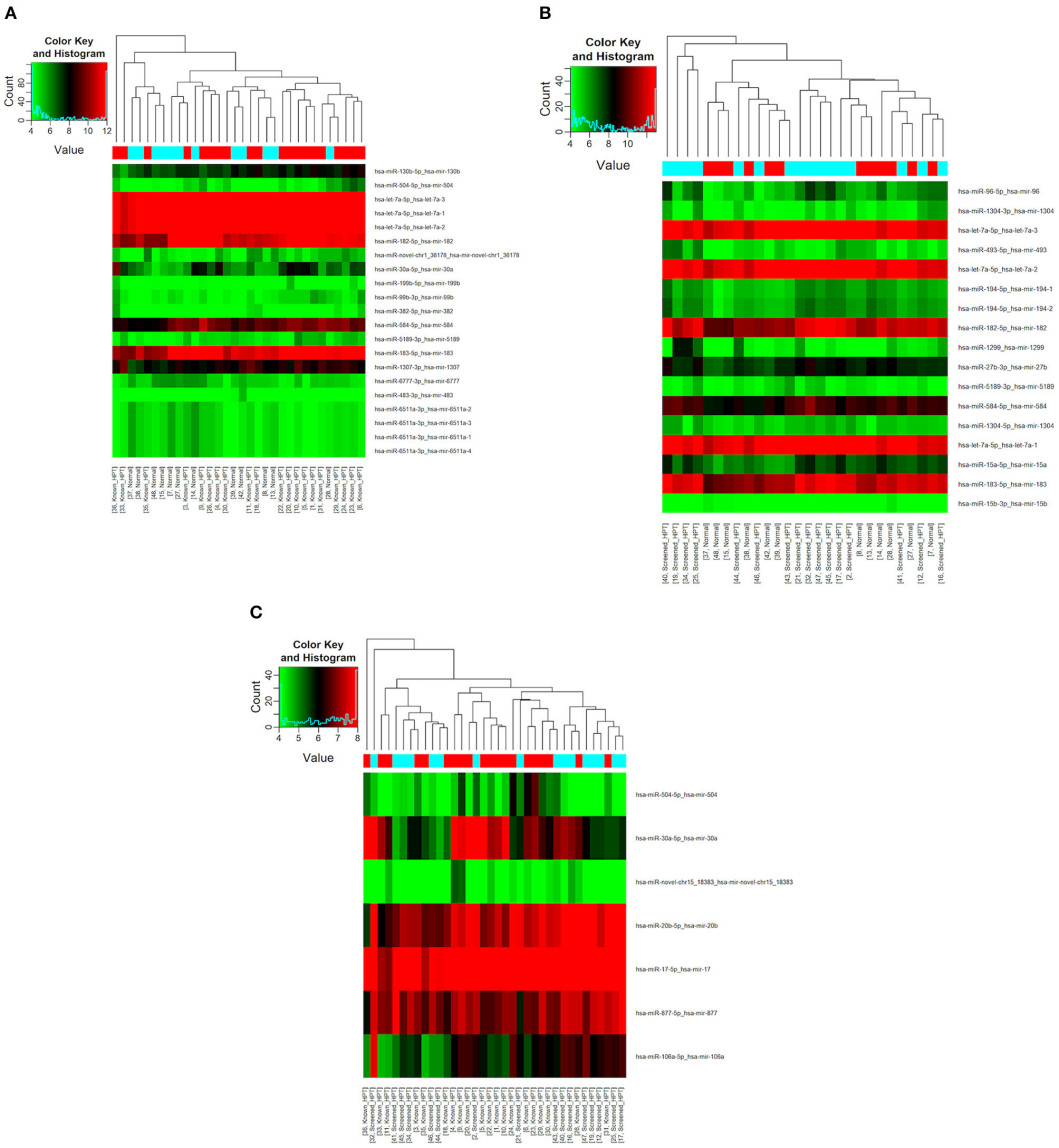
Differential miRNA expression according to HPT status. The heatmap shows all differentially expressed miRNAs at adjusted *p*-value < 0.05. **(A)** Normotensive vs. known HPT; **(B)** Normotensive vs. screen-detected HPT; **(C)** Known HPT vs. screen-detected HPT.

**Figure 2:**
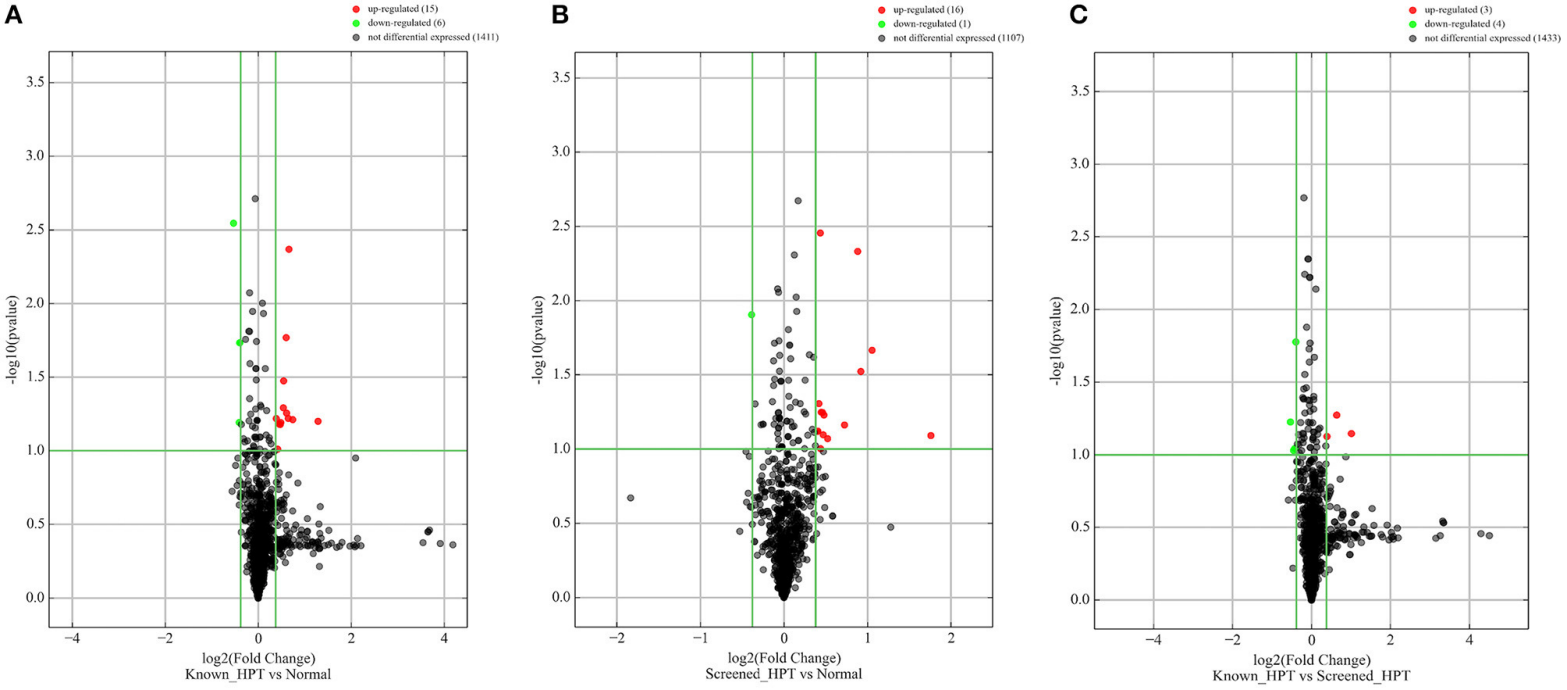
Differentially expressed pre-miRNAs in comparison between blood pressure groups. **(A)** Is a comparison between known hypertensives and normotensives. **(B)** Is a comparison between screen-detected HPT and normotensives. **(C)** Is a comparison between known HPT and screen-detected HPT. Significantly different expression of a pre-miRNA was those in which there was a ≥ 1.3-fold change difference in expression between comparison groups and *p* ≤ 0.1. Red and green dots signify upregulated and downregulated miRNAs, respectively. No differential expression was signified with a black dot.

All 48 samples met the quality control standards. We generated Heat Map and Unsupervised Hierarchical Clustering on all miRNAs that were expressed in at least one sample, to produce miRNA or condition trees that would allow us to pick out groups of similar miRNAs. The result of hierarchical clustering on conditions showed a distinguishable miRNA expression profile amongst the groups (Figures 1A–C). For the identification of differentially expressed miRNAs, we computed “fold changes” (i.e., the ratio of the group averages) and *p*-values between each group. MicroRNAs with fold changes ≥ 1.3 and *p*-values ≤ 0.1 were selected as the differentially expressed miRNAs. Based on pre-specified criteria, we then used volcano plots to visualize the significantly differentially expressed pre-miRNAs between the study groups as shown in Figures 2A–C. A total of 30 significantly differentially expressed mature miRNAs were identified at varying expression levels and are summarized in Table 2. Of the thirty differentially expressed miRNAs, two (6.7%) were novel, and whilst one of these novel miRNAs was upregulated in known HPT vs. normotensive, the other was upregulated in known HPT vs. screen-detected HPT. Whilst miR-1299 exhibited the highest fold change of all significantly upregulated miRNAs as seen in screen-detected HPT vs. normotensive (fold change = 3.38, *p* = 0.0812), miR-30a-5p upregulation was greatest in known HPT vs. normotensive (fold change = 2.44, *p* = 0.0631) and known HPT vs. screen detected HPT (fold change = 2.02, *p* = 0.0715; Table 2).

**Table 2:** Dysregulated mature miRNAs in screen-detected and known HPT compared to normotensive participants.

**Mature miRNA**	**miRNA accession number**	**Known HPT vs. normotensive fold change**	***p*-value**	**BH**	**Mature miRNA**	**miRNA accession number**	**Screen-detected HPT vs. normotensive fold change**	***p*-value**	**BH**	**Mature miRNA**	**miRNA accession number**	**Known HPT vs. screen-detected HPT**	***p*-value**	**BH**
				**FDR**					**FDR**					**FDR**
miR-30a-5p	MIMAT0000087	2.44	0.063	0.7403	miR-1299	MIMAT0005887	3.38	0.081	0.8106	miR-30a-5p	MIMAT0000087	2.02	0.072	0.7031
miR-504-5p	MIMAT0002875	1.67	0.062	0.7403	miR-182-5p	MIMAT0000259	2.08	0.022	0.8106	miR-504-5p	MIMAT0002875	1.56	0.053	0.7031
miR-5189-3p	MIMAT0027088	1.58	0.004	0.7403	miR-96-5p	MIMAT0000095	1.89	0.030	0.8106	miR-novel-chr15_18383	miR-novel-chr15_18383	1.31	0.075	0.7031
miR-182-5p	MIMAT0000259	1.57	0.060	0.7403	miR-183-5p	MIMAT0000261	1.84	0.005	0.8106	miR-877-5p	MIMAT0004949	0.76	0.017	0.7031
miR-183-5p	MIMAT0000261	1.53	0.056	0.7403	miR-493-5p	MIMAT0002813	1.65	0.069	0.8106	miR-106a-5p	MIMAT0000103	0.75	0.091	0.7031
miR-1307-3p	MIMAT0005951	1.52	0.017	0.7403	miR-1304-3p	MIMAT0022720	1.44	0.085	0.8106	miR-17-5p	MIMAT0000070	0.73	0.093	0.7031
miR-novel-chr1_36178	miR-novel-chr1_36178	1.46	0.034	0.7403	miR-5189-3p	MIMAT0027088	1.39	0.059	0.8106	miR-20b-5p	MIMAT0001413	0.69	0.060	0.7031
miR-382-5p	MIMAT0000737	1.45	0.051	0.7403	miR-584-5p	MIMAT0003249	1.38	0.080	0.8106					
miR-584-5p	MIMAT0003249	1.4	0.064	0.7403	miR-27b-3p	MIMAT0000419	1.38	0.057	0.8106					
miR-130b-5p	MIMAT0004680	1.39	0.066	0.7403	miR-194-5p	MIMAT0000460	1.36	0.057	0.8106					
let-7a-5p	MIMAT0000062	1.37	0.066	0.7403	miR-15a-5p	MIMAT0000068	1.36	0.099	0.8106					
miR-199b-5p	MIMAT0000263	1.34	0.098	0.7403	miR-1304-5p	MIMAT0005892	1.35	0.004	0.8106					
miR-99b-3p	MIMAT0004678	1.31	0.060	0.7403	let-7a-5p	MIMAT0000062	1.32	0.076	0.8106					
miR-6511a-3p	MIMAT0025479	0.76	0.019	0.7403	miR-15b-3p	MIMAT0004586	0.76	0.013	0.8106					
miR-483-3p	MIMAT0002173	0.75	0.064	0.7403										
miR-6777-3p	MIMAT0027455	0.69	0.003	0.7403										

*A comparison of dysregulated miRNAs between screen-detected HPT and known HPT participants is also shown*.

*BH FDR, Benjamini-Hochberg False Discovery Rate corrected p-value*.

**Figure 3:**
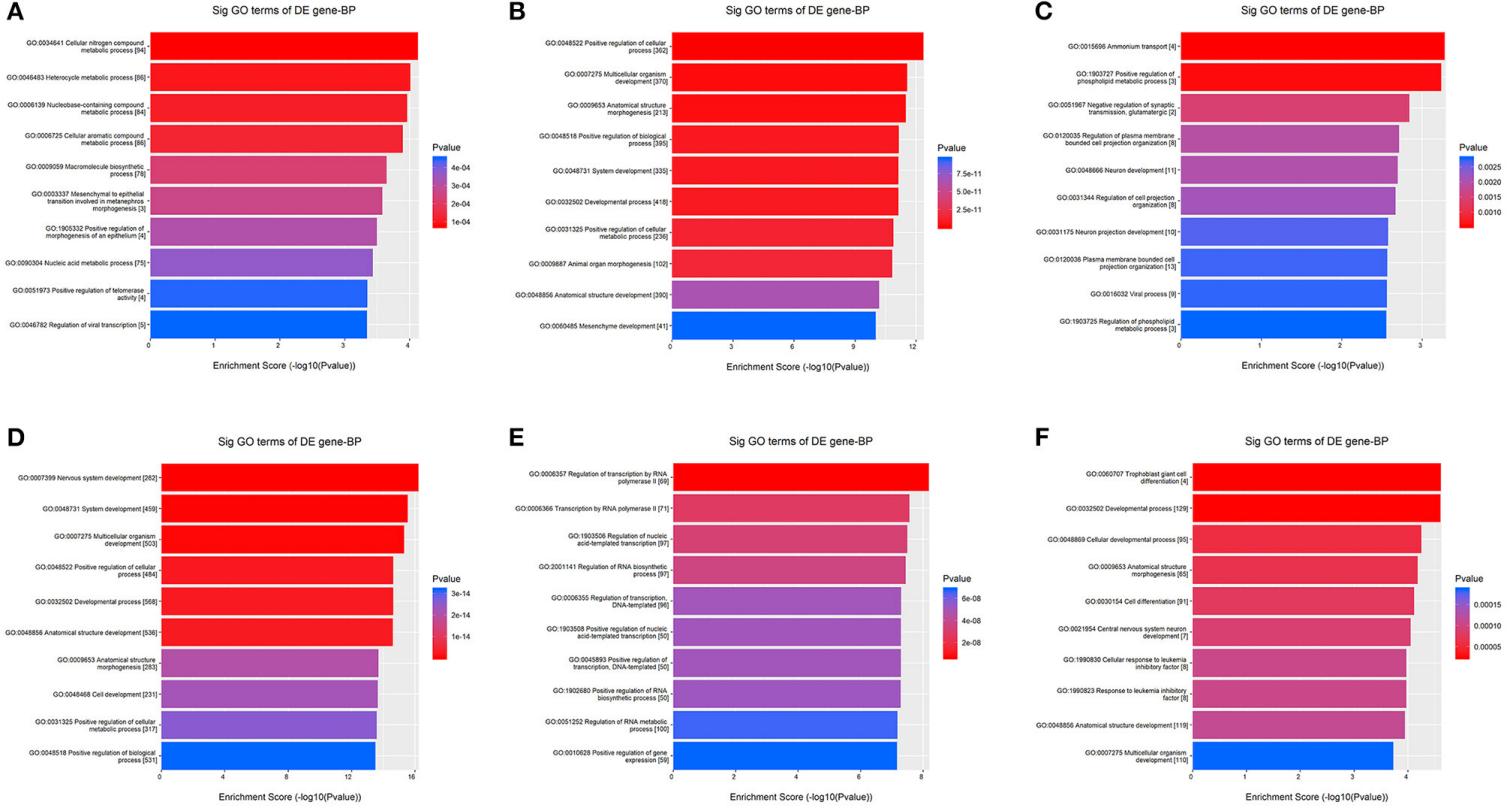
GO analysis—Biological Processes. The bars plots show the top 10 enrichment score values of the significant enrichment terms. X-axis: GOID's enrichment score value; it equals –log10(*p*-value); Y-axis: GO category. **(A)** Upregulated in known HPT vs. normotensive. **(B)** Downregulated in known HPT vs. normotensive. **(C)** Upregulated in screen-detected HPT vs. normotensive. **(D)** Downregulated in screen-detected HPT vs. normotensive. **(E)** Upregulated in known HPT vs. screen-detected HPT. **(F)** Downregulated in known HPT vs. screen-detected HPT.

Kyoto Encyclopedia of Genes and Genomes pathway analysis revealed 84 pathways, five of which are essential for platelet activation, calcium signaling, vascular smooth muscle contraction, vasopressin-mediated water reabsorption and aldosterone synthesis and secretion. Based on GO analyses, we retrieved the biological processes, cellular components and molecular functions of dysregulated miRNAs. In Figure 3, we present the top enrichment scores for biological processes of dysregulated miRNAs in hypertensive vs. normotensive participants.

### Next Generation Sequencing Results Validation

**Figure 4:**
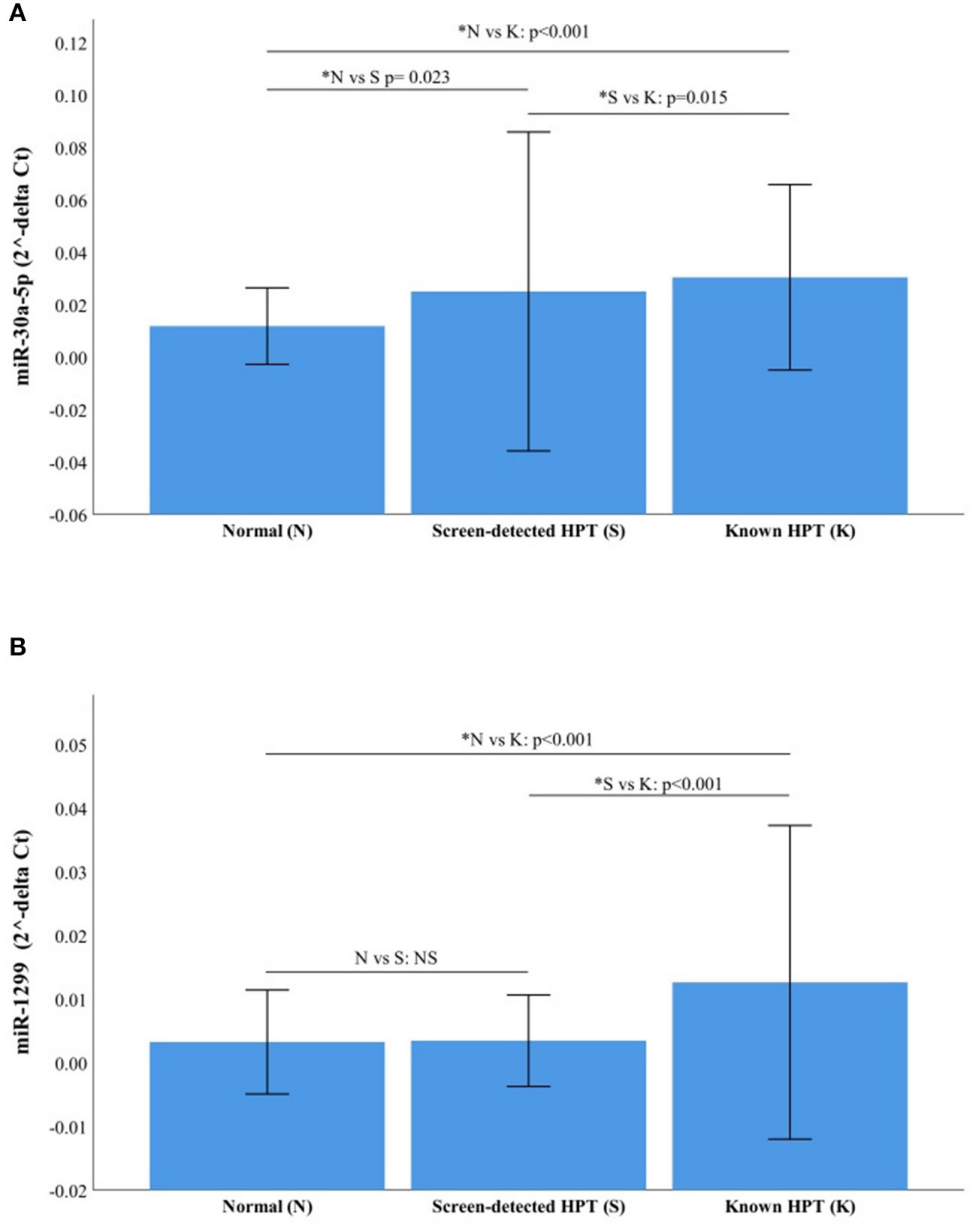
Relative expression of miR-30a-5p and miR-1299. **(A)** miR-30a-5p in normotensive (*n* = 116), screen-detected HPT (*n* = 66) and known HPT (*n* = 124). **(B)** miR-1299p in normotensive, screen-detected HPT and known HPT. Data are represented graphically as the mean ± SD. Not significant (NS) if *p* > 0.05 and significant *p-*value denoted by an asterisk*.

The RT-qPCR data were normalized using miR-16-5p and the raw Ct values, showing its suitability as an endogenous control in our cohort, are shown in Supplementary Figure 1. The two miRNAs with the highest fold change between study groups using NGS were selected for validation with RT-qPCR, namely miR-30a-5p and miR-1299. The relative expressions (2^−ΔCt^) of each target miRNA in the three participant groups are shown in Figure 4. Both miR-30a-5p and miR-1299 were upregulated in known HPT compared to normotensive or screen-detected HPT, *p* = 0.015, whilst miR-30a-5p was also significantly upregulated in screen-detected HPT vs. normotensive, *p* = 0.023. Using the 2^−ΔΔCt^ formula to compute fold changes between two groups, miR-30a-5p expression was 2.58-fold higher in known HPT vs. normotensive and 1.69-fold higher vs. screen-detected HPT. In screen-detected HPT, miR-30a-5p expression was 1.52-fold higher when compared to the normotensives. As for miR-1299, there was a 3.93-fold and 2.78-fold higher expression in known HPT vs. normotensives and screen-detected HPT, respectively. However, there was not a great difference in the expression of miR-1299 between screen-detected HPT vs. normotensive as shown by the 1.41-fold difference in expression.

### Multivariable Regression Analysis

The results of multivariable regression analysis are shown in Supplementary Table 1. For miR-30a-5p, the crude odds ratio was 1.31 [95% confidence interval (CI): 1.13–1.51, *p* < 0.001] for screen-detected HPT, whilst for known HPT, the OR was 1.30 (95% CI: 1.13–1.49, *p* < 0.001). When the models were adjusted for different variables, the expression of miR-30a-5p remained significantly associated with both screen-detected and known HPT (*p* ≤ 0.019). With regard to miR-1299, the crude odds ratio was 0.80 [95% confidence interval (CI): 0.54–1.20, *p* = 0.284] for screen-detected HPT, whilst for known HPT, the OR was 1.11 (95% CI: 0.96–1.30, *p* = 0.164). There was no significant association between screen-detected and known HPT with miR-1299 expression for all tested models (*p* ≥ 0.134). In this cohort of participants, differences in age, sex, diabetes status, BMI, total cholesterol and trigylerides between normotensives and hypertensives (screen-detected and known) did not significantly impact the expression of both miR-30a-5p and miR-1299.

## Discussion

To our knowledge, no study has been conducted on miRNA expression in relation to HPT in populations from Africa. Using NGS, we identified 30 (including two novel) mature miRNAs that were differentially expressed in 48 South African women with either screen-detected or treated HPT. These miRNAs were associated with pathways such as platelet activation, calcium signaling and vascular smooth muscle contraction pathways which are particularly important in cardiovascular pathogenesis (18–20). Two miRNAs, namely miR-1299 and miR-30a-5p were the most significantly dysregulated in hypertensive individuals and this was validated using RT-qPCR in 311 study participants, confirming the miRNA sequencing results while yielding new findings. Multivariable regression analysis showed that in our cohort, differences in age, sex, diabetes status, BMI, total cholesterol and trigylerides had no significant effect on the expression of miRNAs. Furthermore, the significant relationship between miR-30a-5p expression and screen-detected and known HPT was demonstrated.

Several studies have reported on a number of dysregulated miRNAs in HPT using different tissues, but results remain inconsistent (21–25). A study similar to ours reported 27 dysregulated miRNAs in a sample of 13 individuals with HPT (21), although the miRNAs were not similar to ours. A recurring theme within these miRNA profiling studies in HPT is the inter-study inconsistency of findings. For example, expression of various miRNAs such as miR-21, miR-145-5p, miR-155-5p, miR-181a (26–34) that had been previously associated with BP and HPT were not found in this study. We suspect this may partially be attributed to the diverse genetic makeup of Africans and in particular, our study participants whose heterogeneous genetic makeup comprises 32–43% Khoisan, 20–36% Bantu-speaking Africans, 21–28% European, and 9–11% Asian ancestry (35). Furthermore, differences in the methods used could account for the discordance in inter-study findings as the tissue specific nature of some miRNAs has been previously described (36). In our study, discordant results with regards to miR-1299 were evident between NGS and RT-qPCR. Other studies have employed the candidate miRNA approach and reported on miRNAs that have not necessarily been identified using microarrays or sequencing, highlighting the need for more studies employing the same methodologies and experimental designs and standardized sample preparation before these miRNAs can be utilized as new biomarkers.

In a previous study, miR-30 was down-regulated in the plasma of patients with essential HPT (37). In contrast, our findings using both sequencing and RT-qPCR showed an upregulation of miR-30a-5p in both screen-detected or known hypertensive (on antihypertensive treatment) individuals. This difference in expression may be partially explained by the differences in the sample type used for analysis. Whilst our study utilized whole blood (composed of plasma, red blood cells, platelets and white blood cells) for total miRNA expression, the other study made use of plasma (cell-deficient). Pre-analytical sample manipulation using centrifugation, which is required for obtaining plasma from whole blood, affects miRNA expression profiles, as it removes from the plasma, cell-specific miRNAs that would otherwise have been detected in whole blood (38). Findings similar to ours were also reported by Huang et al. who demonstrated increased plasma expression of miR-30a in essential and white coat HPT, relative to normotensive participants (39). Overexpression of miR-30a has been reported to interfere with the removal of damaged or dead endothelial cells, promoting atherosclerosis and predisposing individuals to cardiovascular complications, like heart attacks (40). Similarly, other miRNAs in the miR-30 family have been associated with cardiovascular diseases and suggestions made that they act as predictors for acute myocardial infarction and heart failure (40, 41). For instance, the overexpression of miR-30b-5p was shown to have a downregulatory effect on a muscleblind-like splicing regulator 1 (MBLN1) transcript in atherosclerosis, possibly playing a role in the regulation of vascular smooth muscle cells VSMCs (42). Another miRNA with interesting results was miR-1299, which was significantly upregulated in screen-detected HPT when compared to the normotensive group, fold change = 3.38. This was also confirmed with RT-qPCR, which indicated a 1.41-fold increase in expression of the miRNA in screen-detected HPT compared to the normotensive group. However, multivariable regression analysis did not indicate a relationship between HPT and the expression of miR-1299. Although miR-1299 is yet to be reported in HPT by other groups, the microRNA has been implicated in Rheumatic Heart Disease (RHD), a common complication of which is pulmonary arterial hypertension (PAH) (43). One study identified miR-1299 as an important role player in suppressing the growth of colon cancer cells *via* downregulation of the signal transducers and activators of transcription (STAT3). STAT3 is as an important component in the heart's adaptation to elevated BP (44, 45). It is possible then that elevated expression of miR-1299, as seen in the hypertensive participants, may be a contributing factor in protecting against cardiovascular events associated with elevated BP levels.

As seen in KEGG analysis, the significantly differentially expressed miRNAs had possible involvement in various pathways relevant to HPT, including vascular smooth muscle contraction, vasopressin-mediated water reabsorption, platelet activation, calcium signaling, and aldosterone synthesis and secretion. Alterations to the vascular smooth muscle cells (VSMCs) phenotype has implications in vascular resistance, BP and HPT and Kontaraki et al. demonstrated differential expression of five miRNAs (miR-1, −21, −133, 143, and −145) previously implicated in the alteration of the VSMC phenotype (34). Water retention is also essential in BP regulation and fluid volume maintenance and various miRNAs have been implicated in these processes. Through repression of the methyl CpG binding protein 2 (*Mecp2*) gene and MeCP2 protein, miR-132 regulates vasopressin synthesis and as such, fluid retention (46), whilst miR-32 and −137 regulate water retention by targeting kidney water channels controlled by vasopressin (47, 48). Dysregulations in aldosterone production or secretion pathways may be a risk for the development of HPT and aldosterone production is reduced due to miR-24 targeting of mRNA from the *CYP11B2* gene (49), whilst angiotensin II-mediated overexpression of miR-21 leads to increased aldosterone secretion (50). Dysregulation of calcium signaling leads to altered responses by the vasculature, a common characteristic in HPT (51). In a murine model, Wu et al. demonstrated the regulation of calcium signaling in the kidney by the miR-30 family (52), whilst another study reported miR-214 as a regulator of the calcium pathways through repression of mRNA encoding the sodium-calcium exchanger protein, Ncx1 (53).

Our study had some limitations. Firstly, we did not investigate the effect of antihypertensive drugs, which could have likely influenced the differential expression of miRNAs between treated (known HPT) and untreated (screen-detected HPT) hypertensive individuals. For instance, in a murine model of salt-sensitive HPT, a high salt diet was accompanied by reduced expression of miR-27a, miR-29a, and miR-133a. However, Nebivolol prevented the high salt-mediated lower expression of miR-27a, whilst there was complete and partial reversal of high salt-induced miR-29a decrease by Nebivolol and Atenolol, respectively. Both medications were able to prevent a decrease in miR-133a expression (54). Second, in our NGS analysis, miRNA expression screening was done in only 48 female participants. However, the RT-qPCR validation was performed in a larger sample that also included male participants. Lastly, only two of the 30 significantly dysregulated miRNAs as shown by NGS were validated by RT-qPCR.

In conclusion, our study demonstrated miRNA dysregulation in hypertensive individuals and to our knowledge, is the first study to do so in a sub-Saharan population. Based on our findings, we have shown a number of miRNAs, particularly, miR-30a-5p and miR-1299 that could be explored further for a potential prognostic role, or as therapeutic targets.

## Data Availability Statement

The datasets presented in this study can be found in online repositories. The names of the repository/repositories and accession number(s) can be found below: https://www.ncbi.nlm.nih.gov/, PRJNA680302.

## Ethics Statement

This Investigation was based on the Cape Town Vascular and Metabolic Health (VMH) Study, which was approved by the Research Ethics Committees of the Cape Peninsula University of Technology (CPUT) and Stellenbosch University (respectively, NHREC: REC−230 408−014 and N14/01/003). Ethical approval was also obtained for this cross-sectional sub-study from the CPUT Health and Wellness Sciences Research Ethics Committee (CPUT/HW-REC 2019/H7). The study was conducted as per the provisions of the Declaration of Helsinki. All procedures were explained to the participants in their language of choice. Once the participants fully understood their participation, they signed informed consent forms to allow the collection of blood and anthropometric data. The patients/participants provided their written informed consent to participate in this study.

## Author Contributions

TM, RE, and AK: conceptualization and funding acquisition. DM, SR, and CW: methodology. DM and SD: formal analysis. DM and CW: investigation. TM: resources. DM, SR, and SD: data curation. DM: writing—original draft preparation. TM, RE, AK, GD, SR, and SH: writing—review and editing. DM, CW, and SR: validation. DM, SD, and TM: visualization. TM, GD, and SH: supervision. TM and SD: project administration. All authors have read and agreed to the published version of the manuscript.

## Conflict of Interest

The authors declare that the research was conducted in the absence of any commercial or financial relationships that could be construed as a potential conflict of interest.

## Supplementary Material

Supplementary Figure 1 A comparison of the raw Ct-values of the RT-qPCR normalizer, miR-16-5p in the three blood pressure groups. No significant difference in expression was observed between groups. Known HPT vs. normotensives, *p* = 0.083; known HPT vs. screen-detected HPT, *p* = 0.118 and normotensive vs. screen-detected HPT, *p* = 0.710.

Supplementary Table 1 Multivariate regression analysis of miRNAs for the presence of screen-detected and known hypertension.
