# Supplementary figures and images for: MicroRNA Profiles in Normotensive and Hypertensive South African Individuals
Source: Front Cardiovasc Med. 2021 Apr 16;8:645541. doi: 10.3389/fcvm.2021.645541 (PMC8085261; doi:10.3389/fcvm.2021.645541)

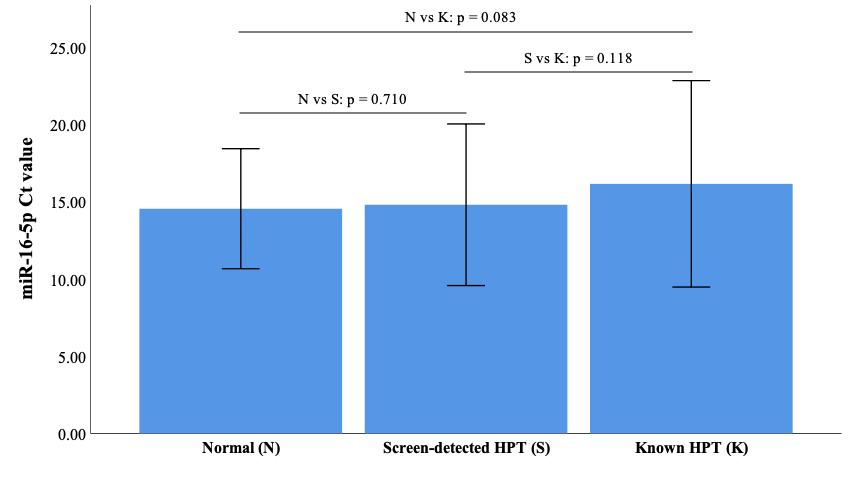

Supplement: Supplementary Figure 1 — A comparison of the raw Ct-values of the RT-qPCR normalizer, miR-16-5p in the three blood pressure groups. No significant difference in expression was observed between groups. Known HPT vs. normotensives, p = 0.083; known HPT vs. screen-detected HPT, p = 0.118 and normotensive vs. screen-detected HPT, p = 0.710. [file Image_1.TIFF]
