# Supplementary material for: MicroRNA Profiles in Normotensive and Hypertensive South African Individuals
Source: Front Cardiovasc Med. 2021 Apr 16;8:645541. doi: 10.3389/fcvm.2021.645541 (PMC8085261; doi:10.3389/fcvm.2021.645541)
Supplement: Supplementary Table 1 — Multivariate regression analysis of miRNAs for the presence of screen-detected and known hypertension. [file Table_1.docx]

**Supplementary Table 1.** Multivariate regression analysis of miRNAs for the presence of screen-detected and known hypertension

|  | **OR** | **95% CI** | ***p-value*** | **OR** | **95% CI** | ***p-value*** |
| --- | --- | --- | --- | --- | --- | --- |
| ***miR 30a-5p**** | | | | | | |
| Model 1 | 1.31 | (1.13; 1.51) | <0.001 | 1.30 | (1.13; 1.49) | <0.001 |
| Model 2 | 1.24 | (1.05; 1.46) | 0.009 | 1.25 | (1.07; 1.47) | 0.005 |
| Model 3 | 1.24 | (1.05; 1.45) | 0.010 | 1.25 | (1.07; 1.47) | 0.006 |
| Model 4 | 1.23 | (1.03; 1.46) | 0.019 | 1.24 | (1.05; 1.47) | 0.013 |
| Model 5 | - | - | - | 1.25 | (0.83; 1.9) | 0.285 |
| ***miR 1299**** | | | | | | |
| Model 1 | 0.80 | (0.54; 1.20) | 0.284 | 1.11 | (0.96; 1.30) | 0.164 |
| Model 2 | 0.75 | (0.50; 1.11) | 0.152 | 0.99 | (0.87; 1.14) | 0.923 |
| Model 3 | 0.73 | (0.48; 1.10) | 0.134 | 0.99 | (0.86; 1.13) | 0.876 |
| Model 4 | 0.76 | (0.51; 1.14) | 0.182 | 1.00 | (0.87; 1.13) | 0.948 |
| Model 5 | - | - | - | 1.00 | (0.76; 1.31) | 0.998 |

Model 1: Crude; Model 2: included age and sex; Model 3: included age, sex and HbA1c; Model 4: included age, sex, BMI, HbA1c, triglycerides, total cholesterol; Model 5: included age, sex, BMI, HbA1c, triglycerides, total cholesterol; duration of disease; *calculated for 0.01-unit increase
